# Amyloid-beta modulates microglial responses by binding to the triggering receptor expressed on myeloid cells 2 (TREM2)

**DOI:** 10.1186/s13024-018-0247-7

**Published:** 2018-03-27

**Authors:** Li Zhong, Zongqi Wang, Daxin Wang, Zhe Wang, Yuka A. Martens, Linbei Wu, Ying Xu, Kai Wang, Jianguo Li, Ruizhi Huang, Dan Can, Huaxi Xu, Guojun Bu, Xiao-Fen Chen

**Affiliations:** 1 Fujian Provincial Key Laboratory of Neurodegenerative Disease and Aging Research, Institute of Neuroscience, Medical College, Xiamen University, Xiamen, 361102 China; 2 Department of Neuroscience, Mayo Clinic, Jacksonville, FL 32224 USA; 3 Neuroscience Initiative, Sanford Burnham Prebys Medical Discovery Institute, La Jolla, CA 92037 USA; 4 Shenzhen Research Institute of Xiamen University, Shenzhen, 518063 China

**Keywords:** β-amyloid, microglia, migration, TREM2, Alzheimer’s disease

## Abstract

**Background:**

TREM2 is an innate immune receptor specifically expressed in microglia. Coding variations in TREM2 have been reported to increase the risk for Alzheimer’s disease (AD) and other neurodegenerative diseases. While multiple studies support a role for TREM2 in microglial recruitment to amyloid plaques, the chemoattractant factor modulating TREM2-dependent microglial responses has not been defined.

**Methods:**

Potential binding of oligomeric amyloid-β 1–42 (oAβ_1–42_) to TREM2 was tested by complementary approaches including solid phase binding, surface plasmon resonance and immunoprecipitation assays. The ability of oAβ_1–42_ to activate TREM2 signaling pathways was examined by analyzing the phosphorylation of Syk and Akt in primary microglia as well as TREM2-mediated signaling in a reporter cell system. Lastly, the functional outcome of oAβ_1–42_-TREM2 interaction was tested by examining impacts on microglial migration in vitro and clustering around oAβ_1–42_-bearing brain areas in vivo.

**Results:**

We found that oAβ_1–42_ bound to TREM2 with high affinity and activated TREM2-dependent signaling pathway. Neither monomeric nor scrambled Aβ bound to TREM2 supporting a specific interaction between oAβ and TREM2. The disease-associated mutations of TREM2 reduced its binding affinity to oAβ_1–42_. Furthermore, we identified several positively charged amino acids within residues 31–91 of TREM2 that were crucial for its interaction with oAβ_1–42_. Importantly, oAβ_1–42_ promoted microglial migration in vitro and clustering in vivo in a TREM2-dependent manner.

**Conclusions:**

Our data establish a critical link between oAβ_1–42_, a major pathological component of AD, and TREM2, a strong genetic risk factor for AD expressed in microglia, and suggest that such interaction contributes to the pathogenic events in AD by modulating microglial responses.

**Electronic supplementary material:**

The online version of this article (10.1186/s13024-018-0247-7) contains supplementary material, which is available to authorized users.

## Background

Alzheimer’s disease (AD) is the most common form of dementia with histopathological hallmarks of amyloid-β (Aβ) plaques and tau neurofibrillary tangles in the brain [1, 2]. While the mechanism by which neurodegeneration occurs in AD is not entirely clear, disturbed Aβ clearance and plaque removal by microglia likely contribute to Aβ accumulation and neuritic dystrophy [3, 4]. As the brain resident innate immune cells, microglia constantly survey the microenvironment and become activated in response to pathogenic components that disrupt the homeostasis of the nervous system [5]. Intriguingly, microglia have been identified to constitute a protective barrier that prevents the outward extension of amyloid fibrils [6, 7]. The involvement of microglia in AD is further supported by recent genome-wide association and genome sequencing studies showing that rare coding variants in genes highly expressed in microglia as risk factors for late-onset AD [8–15]. Single-cell RNA-sequencing in AD mouse model has also identified a novel microglia type that is associated with AD and restricts its development [16]. Therefore, mounting evidence supports a causal role of microglial dysfunction in AD.

TREM2 is a cell surface receptor of the Ig superfamily that is specifically expressed by microglia within the central nervous system (CNS) [17]. Interest in TREM2 stems from its genetic mutations associated with several neurodegenerative disorders [18]. Homozygous missense mutations of *TREM2* including Y38C or T66 M have been identified to be associated with Nasu-Hakola disease (NHD) [19]. The R47H mutation in *TREM2* constitutes one of the strongest single allele genetic risk factors for AD, with an odds ratio similar to that of carrying an apolipoprotein E (*APOE*) ε4 allele [8, 9, 19]. The R62H, D87N and T96 K mutations in *TREM2* were also linked to AD after extensive analyses of *TREM2* polymorphisms [8, 10, 20]. However, the precise effects of TREM2 mutations on AD pathogenesis remain elusive.

TREM2 consists of an ectodomain, a transmembrane region and a short cytoplasmic tail. By coupling with DNAX-activating protein of 12 kDa (DAP12), TREM2 regulates a wide array of functions in microglia including cell survival, proliferation, phagocytosis, and inflammation [21–24]. Recent studies in *Trem2*-deficient AD mouse models have showed contradictory results regarding the amounts of Aβ plaques accumulation [21, 25, 26]. A later study indicated that *Trem2* deficiency has opposing effects on amyloid pathology at early and late stages of AD progression [27]. Nevertheless, it has been consistently reported that *Trem2* deficiency leads to reduced number of microglia around amyloid plaques, allowing greater amount of plaque pathology and related toxicity [26, 28]. However, it remains unknown how TREM2 affects the recruitment of microglia to the vicinity of plaque.

A diverse set of potential TREM2 ligands that are anionic or lipidic in nature have been proposed [29]. Bacteria and poly-anionic molecules were the first ligands proposed for TREM2 [30, 31]. TREM2 was also found to bind macrophages [32], astrocytes [33] and neurons [34]. Recently, TREM2 was shown to bind phospholipids [21], apolipoproteins (including apoE, apoJ and apoA) and lipoprotein particles (including low-density lipoprotein or “LDL”) [35–37]. The binding of apolipoproteins to TREM2 was shown to facilitate either the phagocytosis of apoptotic neurons or the uptake of Aβ by microglia [35, 37]. Interestingly, the AD risk-associated variants, including R47H, R62H and D87N, have reduced affinity to these ligands. While multiple TREM2 ligands have been proposed, the identification of bona fide endogenous TREM2 ligands and the exploration of their physiological and pathological roles remain elusive. In this study, we found that oAβ_1–42_, but not monomeric or scrambled Aβ, bound to TREM2 with high affinity. The disease-associated mutations of TREM2 reduced its binding affinity to oAβ_1–42_. We further identified the specific region of TREM2 encompassing the arginine 47 and 62 residues that is critical for binding to oAβ_1–42_. Functional analyses revealed that oAβ_1–42_ activated TREM2 signaling pathway, promoted microglial migration and clustering in oAβ_1–42_-bearing brain regions via a TREM2-dependent manner. Our data provide mechanistic insight by which microglia recognize Aβ-related pathologies in AD.

## Methods

### Reagents and antibodies

Granulocyte-macrophage colony-stimulating factor (GM-CSF, 415-ML-010), RAGE-Fc recombinant protein (1145-RG-050) and anti-Fc antibody (MAB110) were purchased from R&D systems. Anti-Phospho-Syk (Tyr525/526) (2711 s), anti-total-Syk (13,198 s), anti-Phospho-Akt (Ser473) (4060 s), anti-total-Akt (4685 s) and anti-β-actin antibody (4970 s) were purchased from Cell Signaling Technology. Anti-Aβ antibody (MOAB-2, ab126649) was from Abcam. Unlabeled Aβ_1–42_ (AS-20276), FAM-labeled Aβ_1–42_ (AS-23525-05) and scrambled Aβ_42_ (AS-25382) were from AnaSpec. The mAβ_1–42_ and oAβ_1–42_ were prepared as previously described with minor modification [38, 39]. Dissolved peptides in DMSO (5 mM) were diluted to 100 μM with cold phenol-free F-12 cell culture media (Gibco) and sonicated for 10 min to make mAβ_1–42_ which was used freshly. Part of the peptides were then incubated at 22 °C for 16 h followed by 24 h incubation at 4 °C, centrifuged at 16,000×g for 15 min, and the collected supernatant was used as oAβ_1–42_.

### Western blotting

Cells were lysed with NP-40 lysis buffer (1% NP-40, 50 mM Tris-HCl, pH 8.0, 150 mM sodium chloride containing protease inhibitor mixture from Roche Applied Science) 24 h post transfection. Protein concentrations were determined using the BCA protein assay kit (Thermo Fisher Scientific, 23,225). Equal amounts of samples were subjected to sodium dodecyl sulfate-polyacrylamide gel electrophoresis (SDS-PAGE). Proteins were transferred onto PVDF membranes and probed with specific antibodies. Detection was performed using corresponding secondary antibodies and immunoreactive bands were quantified using ImageJ.

### Immunoprecipitation assay

Two micrograms of oAβ_1–42_ were added into 400 μL of 1% solution of Tween-20 in TBS (1% TBST) with protease inhibitor cocktail. Two micrograms of sTREM2-Fc, sTREM1-Fc or Fc protein was pre-bound to 25 μL of protein A beads (Thermo Fisher Scientific, 20,334). The beads were then incubated with oAβ_1–42_ in 1% TBST at 4 °C overnight. Beads were washed 5 times with 1% TBST for 3 min, resuspended in 20 μL 2 × SDS loading buffer and subjected to electrophoresis on 4–12% Bis-Tris NuPAGE precast gels (Thermo Fisher Scientific, NP0322PK2).

### Immunofluorescence staining

Human embryonic kidney 293 T cells (HEK 293 T) were transfected with pmCherry-N1, pmCherry-TREM1 or pmCherry-TREM2 plasmid. Twenty-four hours after transfection, cells were washed 3 times with DMEM and incubated with 1.0 μM FAM-labeled oAβ_1–42_ for 2 h at 4 °C. After 3 washes with PBS, cells were fixed with 4% paraformaldehyde for 15 min. Cells were then stained with DAPI for 3 min and then washed twice with PBS for 15 min. Coverslips were mounted on the glass slide using antifade reagent (Thermo Fisher Scientific, P36935) and observed using NIKON A1R Plus confocal microscope.

### Expression and purification of sTREM2-Fc fusion proteins

The human TREM2 extracellular domain tagged with Fc that carries different fragments and mutations were cloned and purified from the conditioned medium of HEK 293 T cells as previously described [35]. Purified proteins were quantified with the BCA protein assay kit and the purity was determined on silver-stained SDS-PAGE gels.

### Solid phase binding assay

A 96-well plate (Corning Incorporated) was coated with 100 nM oAβ_1–42_ in PBS overnight at 4 °C. Wells were washed with 0.05% PBST and blocked with 4% BSA for 1 h at 37 °C. Purified Fc or sTREM2-Fc proteins diluted in PBST containing 0.5% BSA were added and incubated for 30 min at 37 °C. After washing, the bound proteins were detected with biotinylated-anti-Fc antibody for 1 h at 37 °C. Plates were washed and then incubated with avidin-HRP for 30 min at 37 °C, washed again, and developed with TMB substrate solution (Sigma Aldrich, T5569), and read at 620 nm.

### Mouse primary microglial culture

All animal experiments were conducted in compliance with the protocols of the Institutional Animal Care and Use Committee at Xiamen University. *Trem2*-knockout mice (KO, on C57BL/6 N background) and wild-type (WT) C57BL/6 N mice were obtained from the UC Davis Knockout Mouse Project (KOMP) repository as described previously [35]. Primary microglial cultures were prepared as previously described [35, 40]. WT or *Trem2*-KO mice at postnatal day 1–3 were used to prepare mixed glial cultures. Cells were plated onto poly-lysine coated flasks and grown in fresh DMEM (Gibco) supplemented with 10% FBS (Gibco). Medium was changed after 3 days to that contains 25 ng/mL GM-CSF and 10% FBS. Primary microglial cells were harvested after 10–12 days in culture and once every 3 days thereafter (up to two harvests).

### Transwell assay

Cell migration assay was performed with Transwell cell culture inserts comprised of two chambers separated by an 8.0 μm polycarbonate membrane (Costar, 3422). Primary microglial cells (5 × 10^4^) suspended in serum-free DMEM were added to the upper chamber of the inserts with serum-free DMEM in the bottom chamber. After 30 min incubation at 37 °C, the bottom medium was replaced with DMEM containing vehicle, 0.5 μM oAβ_1–42_ or 10% FBS. After 20 h, cells remained on the upper surface of the membrane were removed by scraping with a cotton swab. Cells that migrated through the membrane were fixed with 4% paraformaldehyde, stained with hematoxylin and eosin, and counted under Nikon inverted microscope.

### TREM2 reporter assay

BWZ thymoma reporter cells (WT and TD4) were a generous gift from Dr. William E. Seaman (UCSF). BWZ cells express lacZ under control of the promoter for nuclear factor of activated T cells (NFAT) [41]. The TD4 cells are stably transfected to co-express TREM2 and DAP12 [34]. TD4 or WT cells were seeded in 96-well plates at 10^5^ cells/well in RPMI media supplemented with 10 ng/mL phorbol 12-myristate 13-acetate (PMA). Ionomycin was added to a final concentration of 1 μM to serve as a positive control. Reporter cells were stimulated with different concentrations of oAβ_1–42_, mAβ_1–42_ or scAβ_42_ for 16 h at 37 °C, washed once with PBS, and lysed in a buffer containing 100 mM 2-mercaptoethanol, 9 mM MgCl_2_, 0.125% NP-40, and 0.3 mM chlorophenol red-β-D-galactopyranoside. After 4 h at 37 °C, absorbance was measured at 595 nm, with correction for background absorbance at 630 nm.

### Surface Plasmon Resonance assay

Surface Plasmon Resonance (SPR) analysis was performed at 25 °C using the Biacore T200 (GE Healthcare). Purified sTREM2-Fc and Fc protein were immobilized onto Biacore CM5 (GE Healthcare, BR-1005-30) chip using amine coupling kit (GE Healthcare, BR-1000-50). oAβ_1–42_, mAβ_1–42_ or scAβ_42_ was tested with a gradient concentration of 1.25 μM, 2.5 μM, 5.0 μM and 10.0 μM respectively.

### Stereotactic injection of oAβ_1–42_

WT or *Trem2*-KO male mice at 6 months of age were anesthetized and placed in a stereotaxic frame. Investigators were blind to the condition of each animal during the experimental procedure and the quantification and analysis stages. A skin incision was made and holes were drilled at defined x (± 2.0 mm from bregma) and y (− 2.2 mm from bregma) positions. The left and the right hemisphere were injected with FAM-oAβ_1–42_ (a total of 1.5 μg in 3.0 μL) or vehicle at 0.30 μL/min with z-depths of 2.0 mm, respectively. Before being withdrawn slowly, the syringe was left in place for 10 min after each injection. Four or sixteen hours after injection, mice were anesthetized and perfused with ice-cold PBS. Brains were fixed in 4% PFA overnight at 4 °C, transferred to 30% sucrose for 48 h before embedded for cryostat sectioning.

### Immunohistochemistry and microscopy

Twelve-μm thick cryosections were washed in PBS for 15 min, permeabilized in 5% normal donkey serum and 0.2% Triton X-100 for 1 h, followed by 48 h’ incubation with Iba1 antibodies at 4 °C. The cryosections were washed with PBS for 30 min and treated with the Alexa-fluorophore-conjugated secondary antibodies for 2 h at RT. Sections were stained with DAPI (5 μg/mL), washed and mounted with anti-fade reagent. For confocal microscopy, 6 μm z-stacks (consisting of 13 optical slices of 0.5 μm thickness) were acquired using a NIKON A1R Plus confocal microscope.

### Statistics

Data were obtained from at least three independent experiments, and presented as mean ± standard deviation (SD). Statistical analyses were performed using Graphpad Prism 5.0 software or SPSS software. Statistical significance was determined by one-way ANOVA or two-way ANOVA when more than two groups were compared and unpaired Student’s *t*-test for single comparisons. *p* < 0.05 was considered significant.

## Results

### Oligomeric Aβ_1–42_ binds to TREM2 and activates TREM2 signaling pathway

**Fig. 1:**
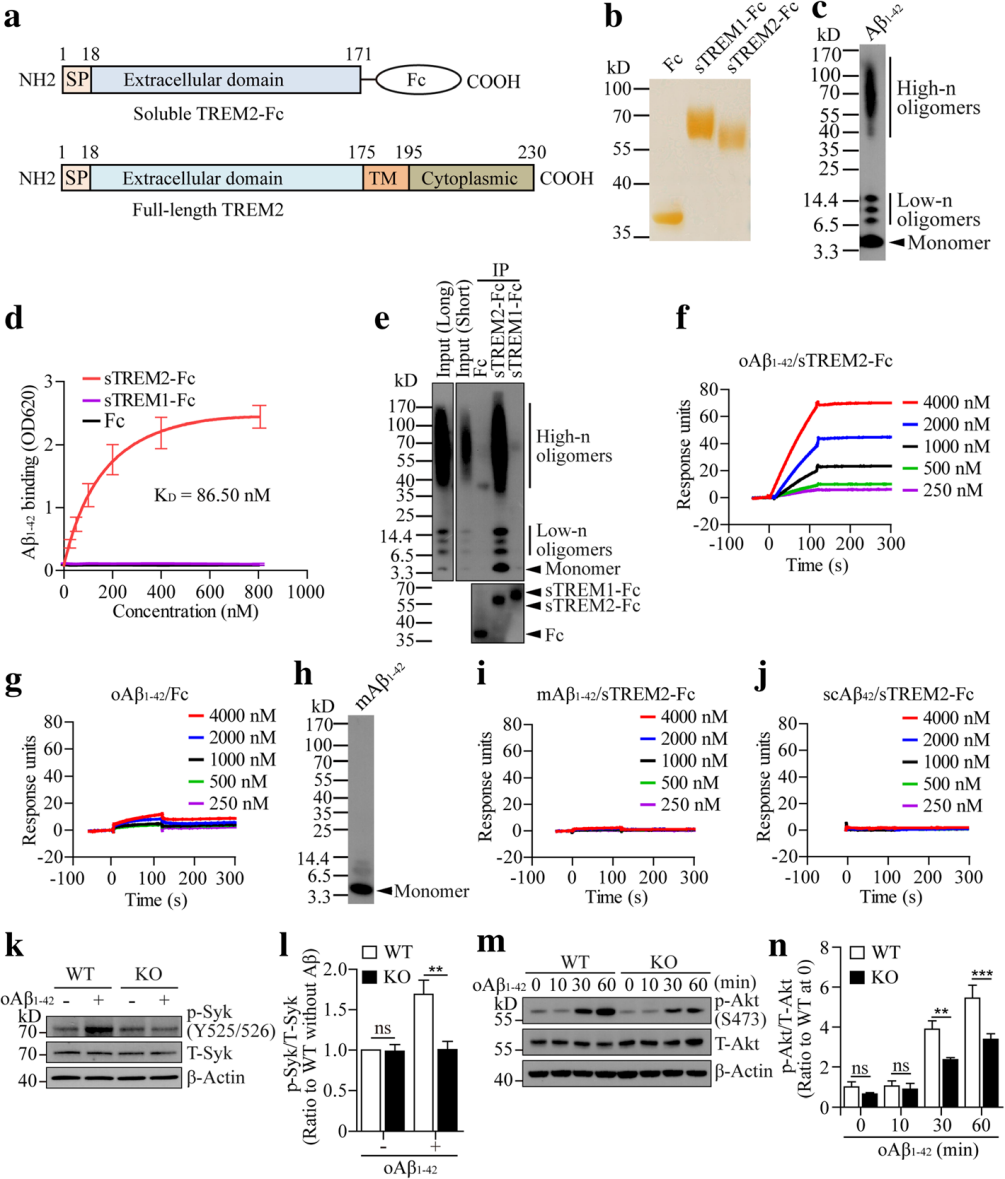
Oligomeric Aβ_1–42_ specifically binds to TREM2 and activates TREM2 signaling pathway**. a** Schematic representation of human TREM2 extracellular domain (sTREM2, amino acid residues 1–171) tagged with human IgG1 Fc. SP: signal peptide. **b** The cDNA encoding sTREM2-Fc, sTREM1-Fc or Fc alone was transfected into HEK 293 T cells. Each protein was purified from the conditioned medium and analyzed by silver stained SDS-PAGE. **c** The prepared oAβ_1–42_ peptides were analyzed by Western blotting using 4–12% Bis-Tris NuPAGE gel. **d** Solid phase binding assay showing the saturation binding curve and equilibrium dissociation constant (K_D_) of oAβ_1–42_ binding to sTREM2-Fc. Fc and sTREM1-Fc served as negative controls (*n* = 3). **e** The Fc, sTREM2-Fc or sTREM1-Fc control was pre-bound to protein A agarose beads and used as baits for immunoprecipitation of oAβ_1–42_. The precipitated products were separated on 4–12% Bis-Tris NuPAGE gel and further subjected to Western blotting. (**f** and **g**) The binding profiles of oAβ_1–42_ to different concentrations of sTREM2-Fc **f** or Fc **g** were generated by SPR assay. **h** The prepared monomeric Aβ_1–42_ (mAβ_1–42_) peptides were analyzed by Western blotting using 4–12% Bis-Tris NuPAGE gel. **i** The binding profiles of mAβ_1–42_ to different concentrations of sTREM2-Fc were generated by SPR assay. **j**The binding profiles of scrambled Aβ_42_ (scAβ_42_) to different concentrations of sTREM2-Fc were generated by SPR assay. **k** Wild-type (WT) or *Trem2*-knockout (KO) microglia were stimulated with 1.0 μM oAβ_1–42_ for 1 h. Cell lysates were analyzed by Western blotting using antibodies specific for either total (T-Syk) or phosphorylated form (p-Syk) of Syk. **l** Quantification of Western blots as ratios of p-Syk/T-Syk. β-Actin was used as an internal control (*n* = 3, two-way ANOVA). **m** WT or *Trem2*-KO microglia were stimulated with 1.0 μM oAβ_1–42_ for the indicated time. Cell lysates at each time point were analyzed by Western blotting using antibodies specific for either total (T-Akt) or phosphorylated form (p-Akt) of Akt. **n** Quantification of Western blots as ratios of p-Akt/T-Akt. β-Actin was used as an internal control (n = 3, two-way ANOVA). Data information: Data represent mean ± SD. **, *p* < 0.01; ***, *p* < 0.001; ns, not significant

TREM2 deficiency was found to reduce the number of microglia clustering around Aβ plaques [21, 25, 26], suggesting a role of TREM2 in bridging the interaction between microglia and plaque. This prompts us to hypothesize that Aβ aggregates serve as chemoattractant for microglia by binding to cell surface TREM2. To test this hypothesis, we used a soluble TREM2-Fc (sTREM2-Fc) construct that consists of the extracellular domain of TREM2 spanning the N-terminal amino acids 1–171 fused to the Fc region of human IgG1 (Fig. 1a) as previously described [42]. The fusion protein was purified from the conditioned medium of transfected human embryonic kidney 293 T (HEK 293 T) cells (Fig. 1b). The extracellular domain of another TREM family member TREM1 fused to Fc (sTREM1-Fc) was also purified and served as a control (Fig. 1b). Soluble oAβ_1–42_ was assembled from synthetic Aβ peptides as previously described [38, 39] and exhibited heterogeneous distribution as low-n and high-n oligomers (Fig. 1c). In the solid phase protein binding assay, we found a specific binding of oAβ_1–42_ to sTREM2-Fc with a K_D_ of 86.50 nM, but not to the Fc region alone or sTREM1-Fc (Fig. 1d). Furthermore, in vitro immunoprecipitation assay revealed that soluble oAβ_1–42_ species in the form of both low-n and high-n aggregates were captured by bead-bound sTREM2-Fc but not sTREM1-Fc or Fc alone control (Fig. 1e), indicating a specific interaction between Aβ oligomers and sTREM2. Consistent with the binding of synthetic Aβ to TREM2, the endogenous Aβ from the brain lysates of 5xFAD amyloid mice model also bound to TREM2 immobilized on beads (Additional file 1: Figure S1A). As a negative control, endogenous Aβ was not detected when TREM1 was used as bait. Therefore, TREM2 specifically interacts with both synthetic and in vivo brain-derived Aβ. Co-localization between TREM2 and oAβ_1–42_ on the cell surface was observed when HEK 293 T cells were transfected with TREM2-Cherry and incubated with FAM-oAβ_1–42_; whereas, co-localization was not observed between TERM1 and oAβ_1–42_ (Additional file 1: Figure S1B and Additional file 1: Figure S1C). The interaction between TREM2 and oAβ_1–42_ was further confirmed by a surface plasmon resonance (SPR) assay which showed a dose-dependent binding of oAβ_1–42_ to sTREM2-Fc fusion protein but not Fc alone (Fig. 1f and g). Since our oAβ_1–42_ was prepared without separation of monomeric Aβ_1–42_ (mAβ_1–42_), we further tested whether mAβ_1–42_ binds to TREM2. An SPR assay with mAβ_1–42_ was performed and showed that mAβ_1–42_ had minimal binding to TREM2 (Fig. 1h and i). The lack of interaction between scrambled Aβ_42_ (scAβ_42_) and sTREM2-Fc further confirmed the specific binding of oAβ_1–42_ to sTREM2-Fc (Fig. 1j). However, we did not include the characterization of fibrillar Aβ in the binding assay due to its structural heterogeneity, limited solubility, and sticky nature. The binding efficiency of oAβ_1–42_ to sTREM2-Fc was compared to that of the receptor for advanced glycosylation endproducts (RAGE), a microglial surface receptor that has been reported to induce microglial activation and chemotaxis upon binding to soluble Aβ [43]. We found TREM2 showed a higher binding affinity for oAβ_1–42_ than RAGE (Additional file 1: Figure S1D), supporting a potentially predominant role of TREM2 in mediating microglial-amyloid interaction. Taken together, our data revealed a specific interaction between TREM2 and oAβ_1–42_, supporting oAβ_1–42_ as a novel ligand for TREM2.

To determine if oAβ_1–42_ can functionally engage TREM2 and initiate intracellular signaling, we utilized TREM2 reporter cells (TD4) which express both TREM2 and DAP12 and produce β-galactosidase upon TREM2 engagement [34]. As a positive control, ionomycin stimulated the expression of reporter β-galactosidase in both wild-type (WT) and TREM2 reporter cells (Additional file 1: Figure S2A and Additional file 1: Figure S2B). Consistent with oAβ_1–42_ as a specific ligand for TREM2, it selectively activated TREM2 reporter cells but not WT cells in a dose-dependent manner, suggesting the requirement of TREM2 engagement. Consistent with the binding data, neither mAβ_1–42_ nor scAβ_42_ stimulated the reporter activity in TD4 cells. To gain more direct evidence that oAβ_1–42_ stimulates TREM2 signaling, we compared the levels of phosphorylated Syk (p-Syk) and Akt (p-Akt) known to represent TREM2 activation in microglia [44]. The stimulation with oAβ_1–42_ induced significantly higher level of p-Syk in WT but not *Trem2*-KO microglia (Fig. 1k and l). Furthermore, oAβ_1–42_ increased to a greater extent of p-Akt in WT than *Trem2*-KO microglia at both the 30- and 60-min time points (Fig. 1m and n). Taken together, these results indicate that oAβ_1–42_ binds to TREM2 and activates TREM2-dependent signaling pathway in microglia.

### The disease-associated mutations reduce TREM2 binding to oAβ_1–42_

**Fig. 2:**
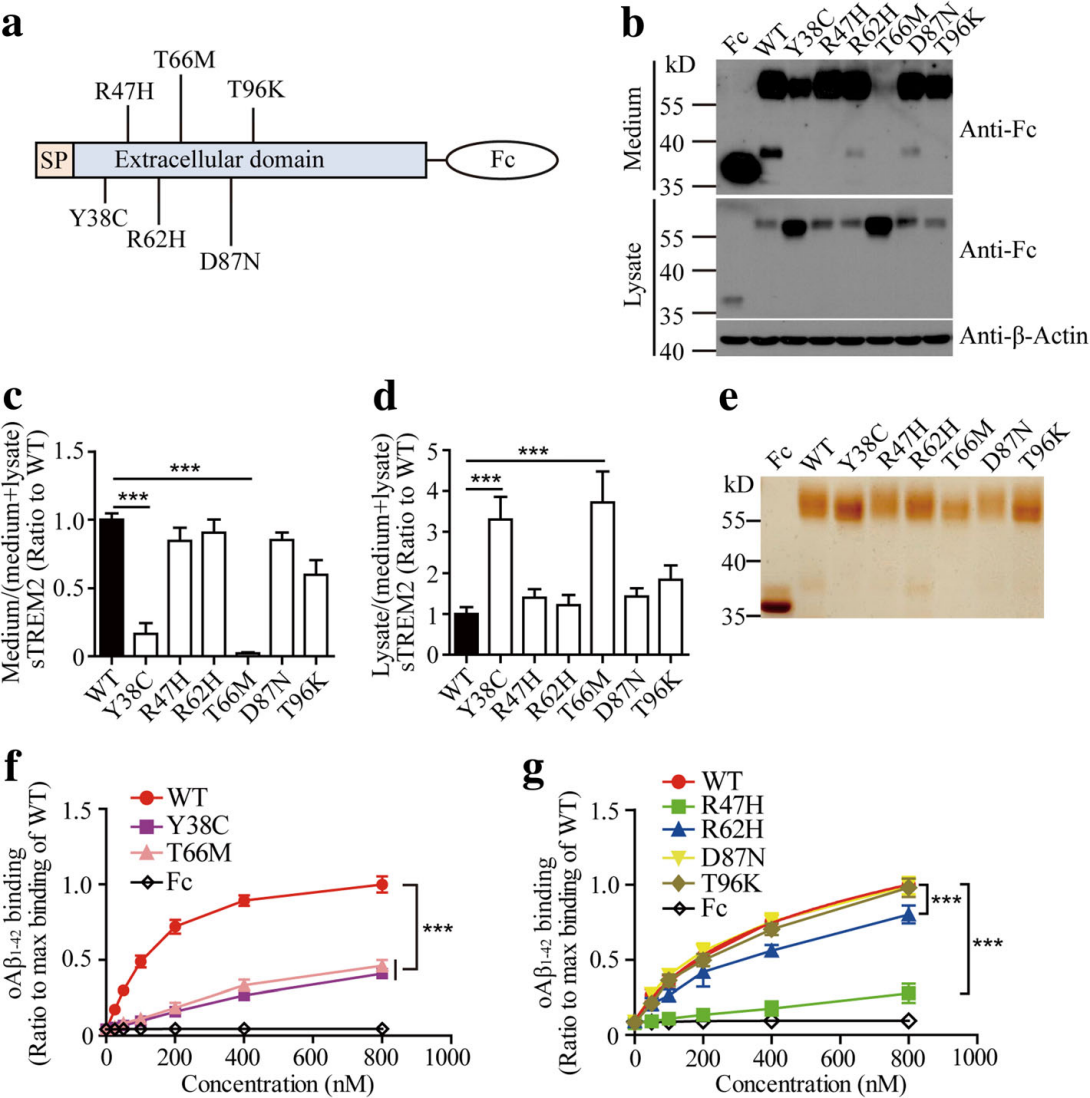
The disease-associated mutations reduce TREM2 binding to oAβ_1–42_. **a** Schematic representation of NHD and AD-associated sTREM2 variants tagged with Fc. **b** Immunoblotting of sTREM2-Fc in the conditioned medium or cell lysate from HEK 293 T cells expressing AD-associated TREM2 mutations (R47H, R62H, D87N and T96 K) or NHD-associated TREM2 mutations (Y38C and T66 M). (**c** and **d**) Bar graphs show the quantification of Western blots from panel B (n = 3, one-way ANOVA). **e** The purified Fc, wild-type sTREM2 WT-Fc (WT) and sTREM2-Fc with disease-associated mutations were analyzed by silver staining. (**f** and **g**) Solid phase binding assay showing the binding affinity of oAβ_1–42_ to WT and different variants of TREM2-Fc (n = 3, two-way ANOVA). Data information: Data represent mean ± SD. ***, *p* < 0.001

The majority of disease-associated mutations are clustered within the putative ligand binding region of TREM2 ectodomain. With this in mind, we investigated how TREM2 variants affect its binding to oAβ_1–42_. We focused on two NHD-causing mutations (Y38C and T66 M) and four AD risk variants (R47H, R62H, D87N and T96 K) by generating sTREM2-Fc constructs carrying corresponding point mutations (Fig. 2a). Expression of Y38C and T66 M mutants in HEK 293 T cells resulted in a strong retention of sTREM2-Fc within the cytoplasm, thereby leading to reduced levels of both proteins in the conditioned medium (Figs. 2b-d). In contrast, none of the four AD risk-associated variants affected surface transport of sTREM2. Nevertheless, all proteins were purified to near homogeneity (Fig. 2e) and subjected to the solid phase protein binding assay using the same amount of each sTREM2-Fc variant. Compared to the wild-type (WT) protein, both NHD mutants exhibited dramatically decreased binding to oAβ_1–42_ (Fig. 2f). Among the four AD risk-associated variants, the D87N and T96 K mutants bound to oAβ_1–42_ with comparable affinity to WT protein (Fig. 2g). However, the R47H variant exhibited a large reduction (72%) in binding to oAβ_1–42_; while the binding of R62H mutant to oAβ_1–42_ was impaired to a lesser degree (20%).

### Residues 31–91 of TREM2, in particular the positively charged amino acids within this region, are crucial for its interaction with oAβ_1–42_

**Fig. 3:**
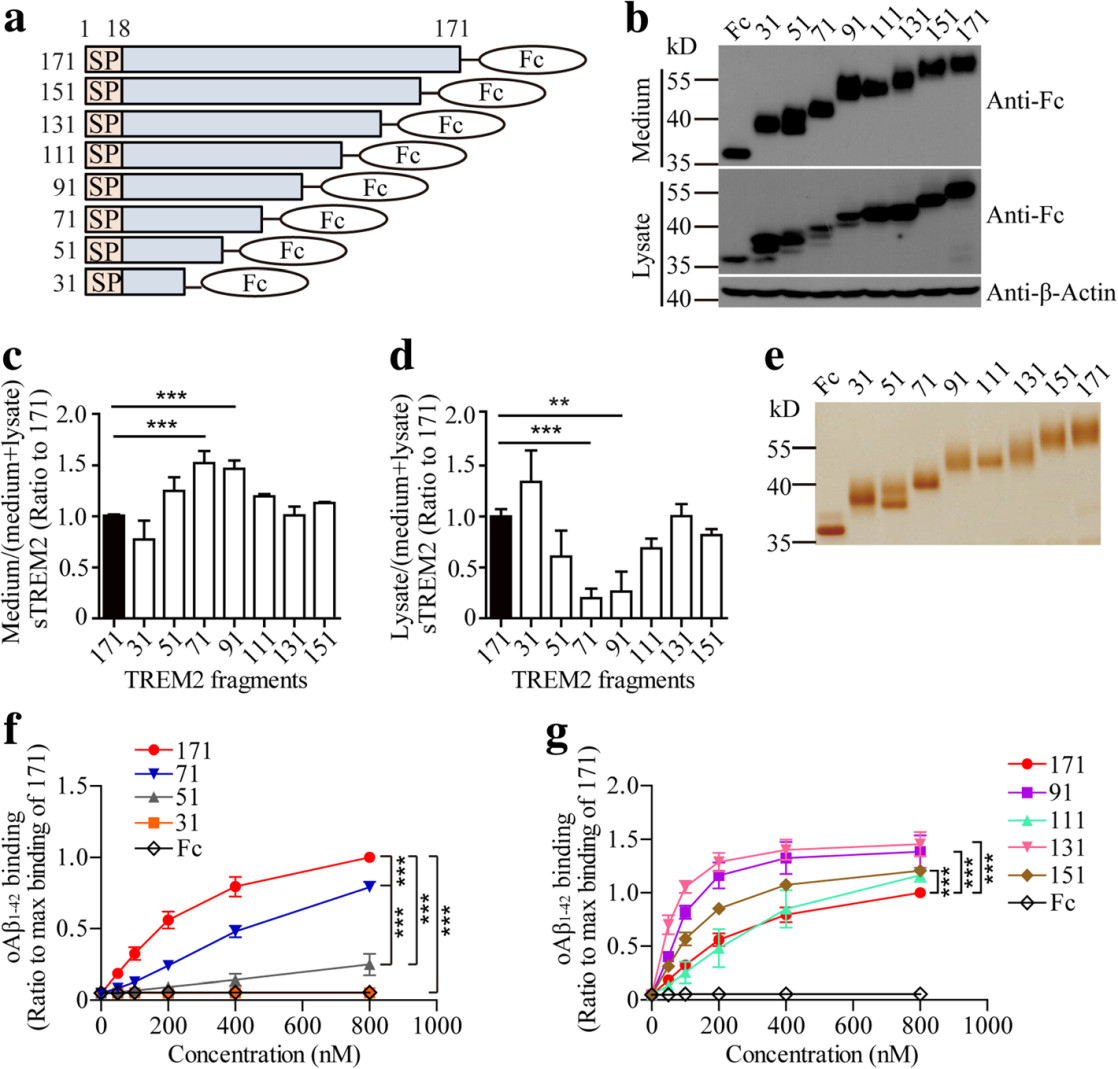
Residues 31–91 of TREM2 are crucial for its interaction with oAβ_1–42_. **a** Schematic representation of different fragments of sTREM2-Fc. **b** Immunoblotting of sTREM2-Fc fragments in the conditioned medium or cell lysate from HEK 293 T cells. (**c** and **d**) Bar graphs showing the quantification of Western blots from panel B (n = 3, one-way ANOVA). **e** The purified Fc and sTREM2-Fc fragments were analyzed by silver staining. (**f** and **g**) Solid phase binding assay showing the binding affinity of oAβ_1–42_ to sTREM2-Fc fragments (n = 3, two-way ANOVA). Data information: Data represent mean ± SD. **, *p* < 0.01; ***, *p* < 0.001

In an attempt to map the critical region on TREM2 for its association with oAβ_1–42_, we constructed a series of C-terminally truncated mutants of sTREM2-Fc (Fig. 3a). Expression of the mutants carrying residues 1–71 and 1–91 resulted in a significantly increased secretion of sTREM2 fragments into the medium (Figs. 3b-d). The truncation mutants were purified and subjected to oAβ_1–42_ binding assay (Fig. 3e). Interestingly, mutants carrying residues 1–31 showed no detectable binding to oAβ_1–42_, while both fragments spanning 1–51 and 1–71 residues exhibited a statistically significant reduction in binding to oAβ_1–42_ as compared to the 1–171 fragment (Fig. 3f). For other truncation mutants, the binding affinity of the 1–111 fragment to oAβ_1–42_ was similar to the 1–171 fragment. However, fragments carrying 1–91, 1–131 and 1–151 residues showed significantly higher affinity to oAβ_1–42_ than the 1–171 fragment (Fig. 3g). Therefore, we conclude that residues 31–91 of TREM2 are necessary for its interaction with oAβ_1–42_.

**Fig. 4:**
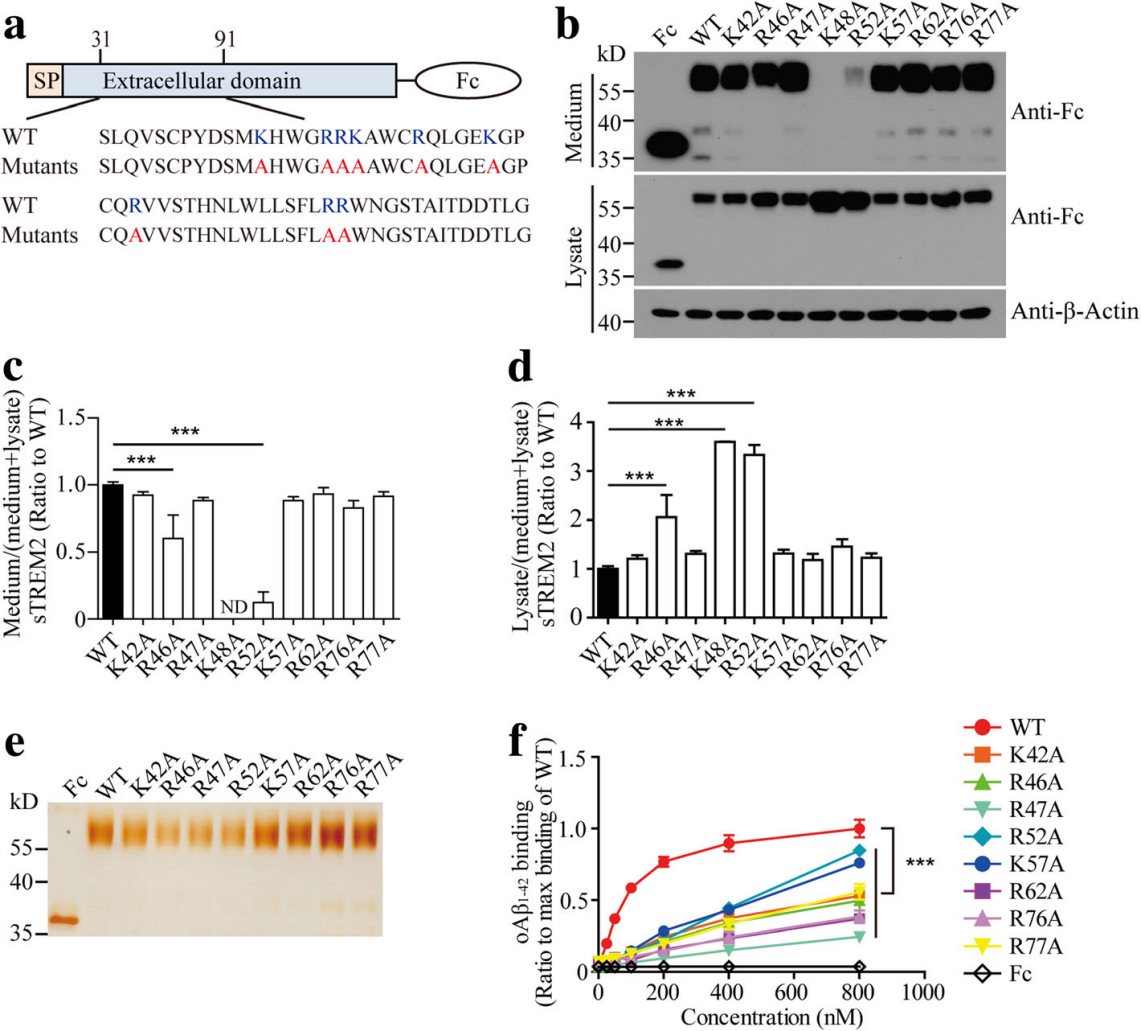
The positively charged amino acids in TREM2 mediate its interaction with oAβ_1–42_. **a** Schematic representation of mutagenesis of positively charged amino acids on sTREM2-Fc. **b** Immunoblotting of sTREM2-Fc mutants in the conditioned medium or cell lysate from HEK 293 T cells. (**c** and **d**) Bar graphs showing the quantification of Western blots from panel B (n = 3, one-way ANOVA). ND: not determined. **e** The purified Fc and sTREM2-Fc mutants were analyzed by silver staining. **f** Solid phase binding assay showing the binding affinity of oAβ_1–42_ to sTREM2-Fc mutants (n = 3, two-way ANOVA). Data information: Data represent mean ± SD. ***, *p* < 0.001

Based on our observation that both R47H and R62H mutants significantly impaired the interaction between TREM2 and oAβ_1–42_, we sought to examine whether the positively charged amino acids (arginine and lysine) within residues 31–91 play important roles in TREM2 binding to oAβ_1–42_. Mutant forms of sTREM2-Fc carrying K42A, R46A, R47A, K48A, R52A, K57A, R62A, R76A or R77A mutation were constructed and expressed in HEK 293 T cells (Fig. 4a). The K42A, R47A, K57A, R62A, R76A and R77A mutants exhibited similar surface transport to WT sTREM2-Fc (Figs. 4b-d). However, the mutations in the forms of R46A, K48A or R52A led to significantly decreased levels of sTREM2 in the medium. Remarkably, the K48A mutation almost completely abolished the secretion of sTREM2 into the extracellular space, therefore preventing the follow-up protein purification for further analysis (Fig. 4e). Interestingly, all the mutations significantly reduced the binding affinity of TREM2 to oAβ_1–42_ (Fig. 4f), indicating that the interaction between TREM2 and oAβ_1–42_ relies on the positively charged amino acids within residues 31-91of TREM2.

### Oligomeric Aβ_1–42_ modulates microglial responses dependent on TREM2 receptor

**Fig. 5:**
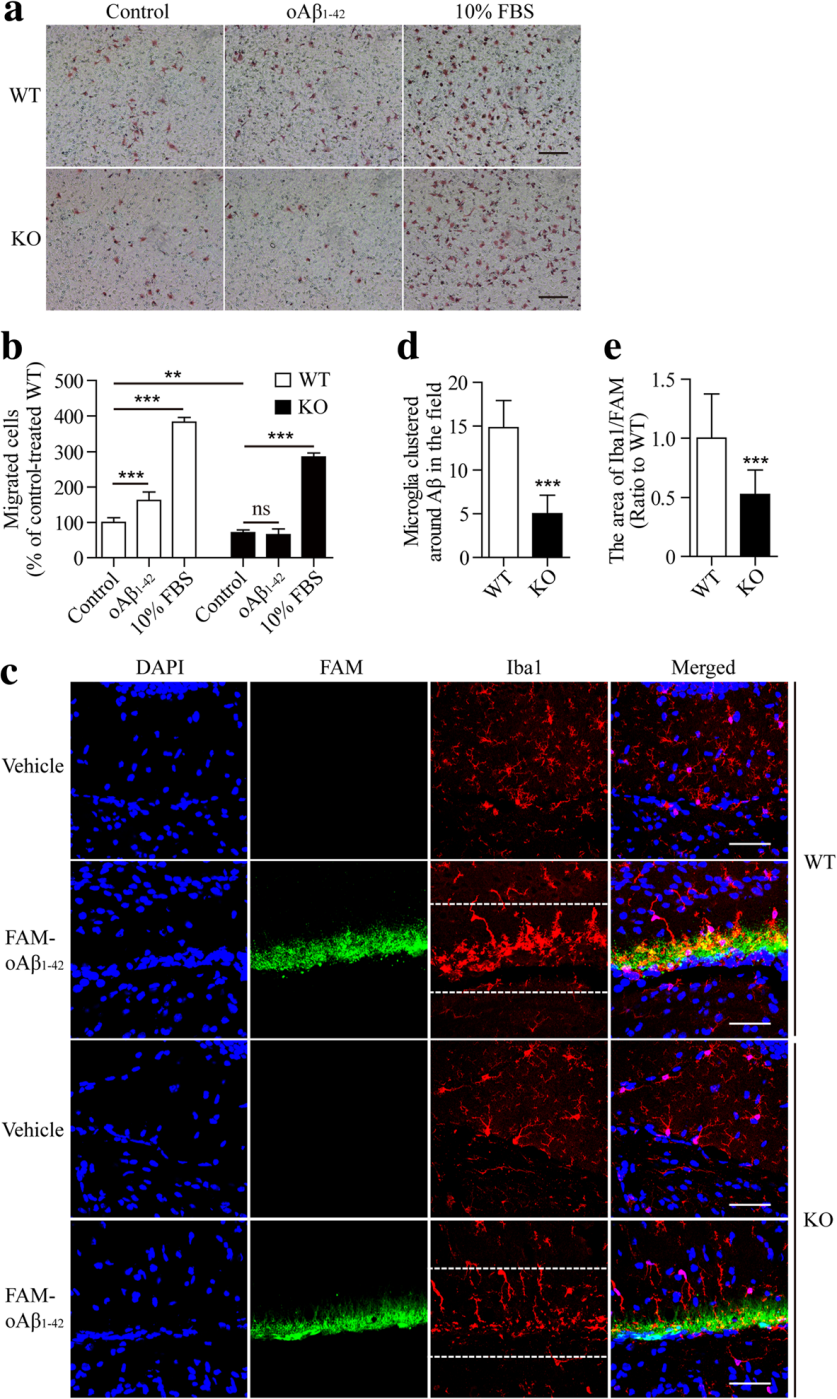
Oligomeric Aβ_1–42_ modulates microglial responses dependent on TREM2 receptor. **a** Primary microglial cells (5 × 10^4^) from WT or *Trem2*-knockout (KO) mice were plated onto transwell chamber inserts. Following 20 h incubation with vehicle, oAβ_1–42_ (0.5 μM), or 10% FBS, cells migrated through the membrane were stained with hematoxylin and eosin and imaged under Nikon inverted microscope. Scale bar, 100 μm. **b** At least 24 different fields from three independent experiments of transwell tests were selected for quantifying the number of migrated cells (two-way ANOVA). **c** WT or *Trem2*-KO (KO) mice brain was harvested after FAM-oAβ_1–42_ injection for 4 h. Coronal sections from these mice were stained with DAPI (blue) for nuclei, and Iba1 (red) for microglia. Representative z stack images of oAβ_1–42_-bearing regions are shown. Scale bar, 50 μm. **d** At least 16 fields (212 μm × 100 μm, marked by white dotted lines in the panel C) from four mice were selected for quantifying the number of microglia (co-stained with DAPI and Iba1) clustered in the oAβ_1–42_-bearing regions (unpaired, Student’s *t*-test). **e** The Iba1-positive area within the white dotted lines in the panel C was normalized to that of FAM-oAβ_1–42_-positive area (unpaired, Student’s *t*-test). Data information: Data represent mean ± SD. **, *p* < 0.01; ***, *p* < 0.001; ns, not significant

Both oAβ_1–42_ and TREM2 have been reported to modulate the migration of microglial cells [45, 46]. We explored whether oAβ_1–42_-induced cell migration in microglia relies on endogenous TREM2. Primary microglia were isolated from both WT and *Trem2*-knockout (KO) mice and subjected to the transwell migration assay in the presence or absence of oAβ_1–42_ (Figs. 5a and b). Consistent with previous reports, the deficiency of *Trem2* led to a significant decrease in cell migration across the transwell membrane [45]. Treatment with oAβ_1–42_ aggregates caused an increase in the migration of WT microglia as compared to the vehicle control. In contrast, oAβ_1–42_ was incapable of inducing migration of *Trem2*-KO microglial cells. Notably, the 10% FBS (Fig. 5a and b) and ATP (Additional file 1: Figure S3A and Additional file 1: Figure S3B) dramatically stimulated the migration of both WT and *Trem2*-KO microglial cells, suggesting that the lack of response to oAβ_1–42_ in *Trem2*-KO microglia is likely specific. To further test whether TREM2 modulates the clustering of microglia around oAβ_1–42_ aggregates in vivo, we stereotactically injected FAM-oAβ_1–42_ into the hippocampi of adult WT and *Trem2*-KO mice and quantified the number of microglia in the vicinity of oAβ_1–42_. A significantly increased number of microglia accumulated around the injected Aβ in WT mouse brain as early as 4 h postinjection; however, the effect was diminished in the absence of *Trem2* (Figs. 5c-e). Similar results were obtained 16 h postinjection (Additional file 1: Figure S3C, Additional file 1: Figure S3D and Additional file 1: Figure S3E), further supporting a key role of TREM2 in microglial clustering around amyloid in vivo. Taken together, our data revealed that oAβ_1–42_ promotes microglial migration in vitro and clustering in vivo in a TREM2-dependent manner.

## Discussion

Human genetic association studies increasingly highlight microglial genes, such as *TREM2*, *CD33*, and *CR1* playing important roles in the pathogenesis of AD [47]. Although mechanistic understanding of TREM2 action remains elusive, TREM2 presumably carries out its function in AD by binding to endogenous ligand(s). The many diverse ligands reported for TREM2 suggest that TREM2 is a highly promiscuous receptor. In this study, we found that oAβ specifically bound to TREM2 with high affinity. The interaction is functionally relevant to the pathogenesis of AD as evidenced by the observation that oAβ triggers microglial migration in vitro and microglial clustering around oAβ-bearing regions in vivo in a TREM2-dependent manner. Therefore, different ligands of TREM2 likely trigger ligand-specific events to accomplish the multi-functions reported for TREM2, in the case of oAβ microglial migration.

Interestingly, the disease-associated mutations of TREM2 reduced its binding affinity to Aβ. Although coding variants of TREM2 are linked to the risk of neurodegenerative diseases, the underlying mechanisms remain unknown. Several recent studies have shed light on how TREM2 missense mutations affect TREM2 function. First, the disease-associated mutations of TREM2 have impaired cell surface expression as reported previously [48, 49] and in our current study. Second, TREM2 missense mutations have been reported to reduce its shedding by ADAM proteases and impair the phagocytic activity of TREM2-expressing cells [48]. Furthermore, AD risk-associated mutations abrogate the function of soluble TREM2 in both suppressing apoptosis and triggering inflammatory responses [42]. Importantly, TREM2 variants exhibited deficits in binding to a wide array of ligands, including phospholipids, apolipoproteins, lipoprotein particles [21, 35–37] and oAβ_1–42_ as reported in the current study. Taken together, the disease-associated mutations of TREM2 likely modify the disease progression via multiple pathways.

Although our current study emphasizes a critical role of membrane-bound TREM2 which associates with DAP12 for downstream signaling, we could not rule out a potential contribution by sTREM2, a shed product of membrane-bound TREM2, that binds to a yet identified microglial receptor [42]. Hence, both full-length TREM2 and sTREM2 might be involved in microglial recruitment to the vicinity of plaques. Although the molecular mechanism underlying each pathway remains to be defined in future, our data suggest that the membrane-bound TREM2 signals through Syk and Akt, while sTREM2 might act on a separate microglial cell-surface receptor [42].

One striking feature of microglia in AD brain is their universal clustering around amyloid plaque. It was consistently reported that the number of microglia around amyloid plaques was reduced in *Trem2*-deficient amyloid mouse models, leading to an increase in less compact plaques [26, 28]. A critical function of microglia is thought to remove Aβ deposits via phagocytosis [50]. Indeed, loss of *Trem2* has been reported to impair the phagocytosis of Aβ by microglia in vitro [48]. Therefore, the physical interaction between TREM2 and Aβ likely regulates microglial mobility, potentially contributing to both the barrier function of microglia to compact the plaques and the phagocytic activity of microglia in removing the plaques. Future studies elucidating the biological function of their interaction might uncover targetable pathways for AD therapy.

## Conclusions

Our current study demonstrates that oAβ binds to TREM2 with high affinity and activates TREM2-dependent signaling pathways in microglia. The absence of TREM2 impairs the oAβ-induced microglial migration in vitro and microglial clustering around oAβ-bearing brain regions in vivo. The identification of Aβ as a novel TREM2 ligand links TREM2 to the most prominent pathological features of AD and provides a critical mechanism by which microglia recognize and react to Aβ pathology.

## Additional file

Additional file 1: **Figure** **S1.** Oligomeric Aβ_1–42_ specifically binds to TREM2. **Figure S2.** Oligomeric Aβ_1–42_ specifically activates TREM2 reporter cells. **Figure S3.** The number of microglia clustered around Aβ is decreased in *Trem2*-KO mice. (PDF 1369 kb)

## Abbreviations

AD: Alzheimer’s Disease
CNS: Central Nervous System
DAP12: DNAX-activating Protein of 12 kDa
GM-CSF: Granulocyte-Macrophage Colony-stimulating Factor
oAβ_1–42_: Oligomeric Amyloid-β 1–42
RAGE: Receptor for Advanced Glycosylation Endproducts
SPR: Surface Plasmon Resonance
sTREM2: soluble TREM2
TREM1: Triggering Receptor expressed on Myeloid Cells 1
TREM2: Triggering Receptor expressed on Myeloid Cells 2
