# Supplementary material for: Amyloid-beta modulates microglial responses by binding to the triggering receptor expressed on myeloid cells 2 (TREM2)
Source: Mol Neurodegener. 2018 Mar 27;13:15. doi: 10.1186/s13024-018-0247-7 (PMC5870375; doi:10.1186/s13024-018-0247-7)
Supplement: Supplementary file 1 — Figure S1. Oligomeric Aβ1–42 specifically binds to TREM2. Figure S2. Oligomeric Aβ1–42 specifically activates TREM2 reporter cells. Figure S3. The number of microglia clustered around Aβ is decreased in Trem2-KO mice. (PDF 1369 kb) [file 13024_2018_247_MOESM1_ESM.pdf]

Supplemental Data

Figure S1

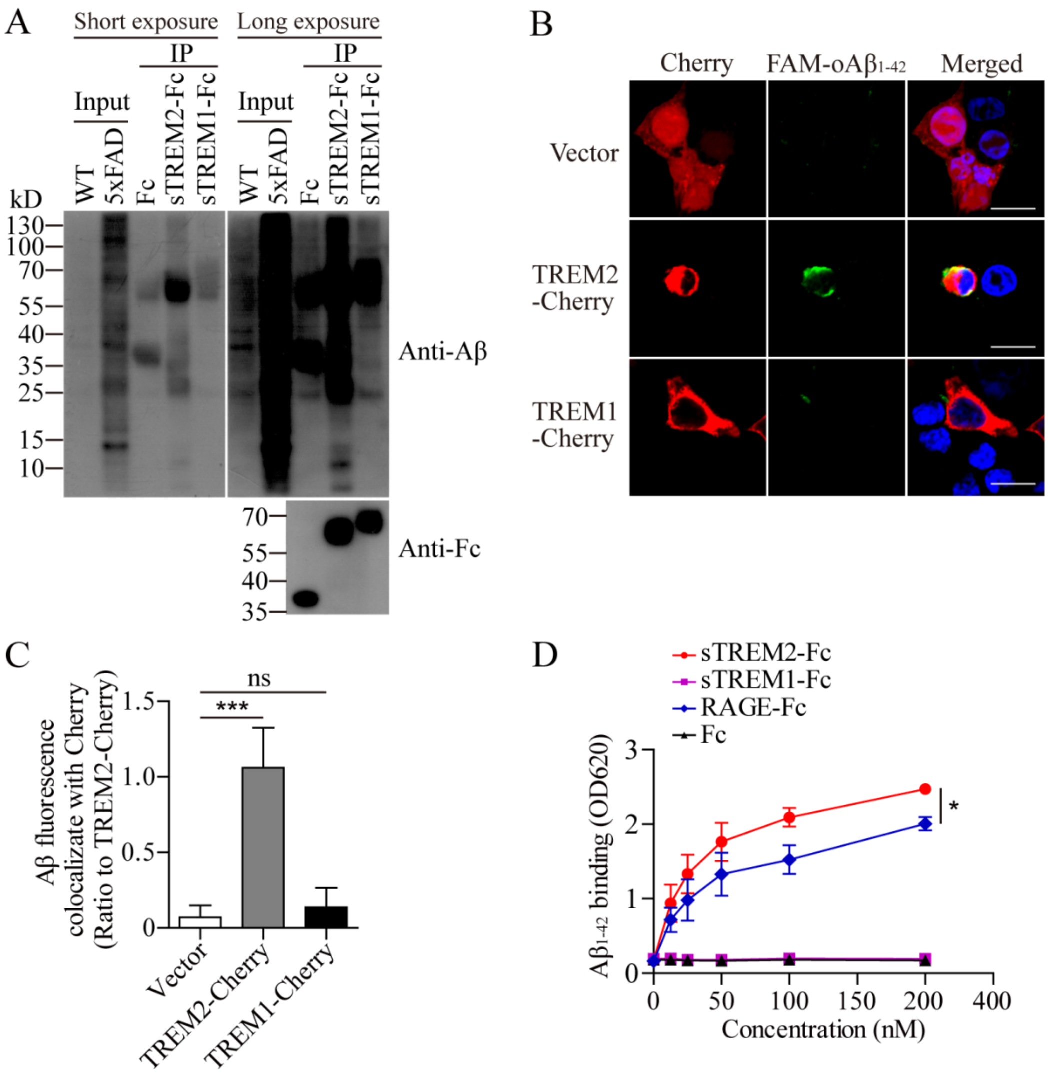

Fig. S1 Oligomeric A$\beta_{1\text{-}42}$ specifically binds to TREM2. (A) The Fc, sTREM2-Fc or sTREM1-Fc was pre-bound to protein A agarose beads and used as baits for immunoprecipitation of Aβ from the brain lysates of 5xFAD mice. The precipitated products were separated on 4-12% Bis-Tris NuPAGE gel and further subjected to Western blotting. Note that sTREM2-Fc co-immunoprecipitated greater amounts of heterogeneous Aβ species compared to Fc or

sTREM1-Fc. (B and C) HEK 293T cells were transfected with pmCherry-N1, pmCherry-TREM1 or pmCherry-TREM2 plasmid and incubated with 1.0 μM FAM-oA$\beta_{1\text{-}42}$ for immunofluorescence analysis (n = 3, one-way ANOVA). Scale bar, 10 μm. (D) Solid phase binding assay showing the binding affinity of oA$\beta_{1\text{-}42}$ to sTREM2-Fc and RAGE-Fc (n = 3, two-way ANOVA). Data information: Data represent mean ± SD. *, $p < 0.05$; ***, $p < 0.001$; ns, not significant.

Figure S2

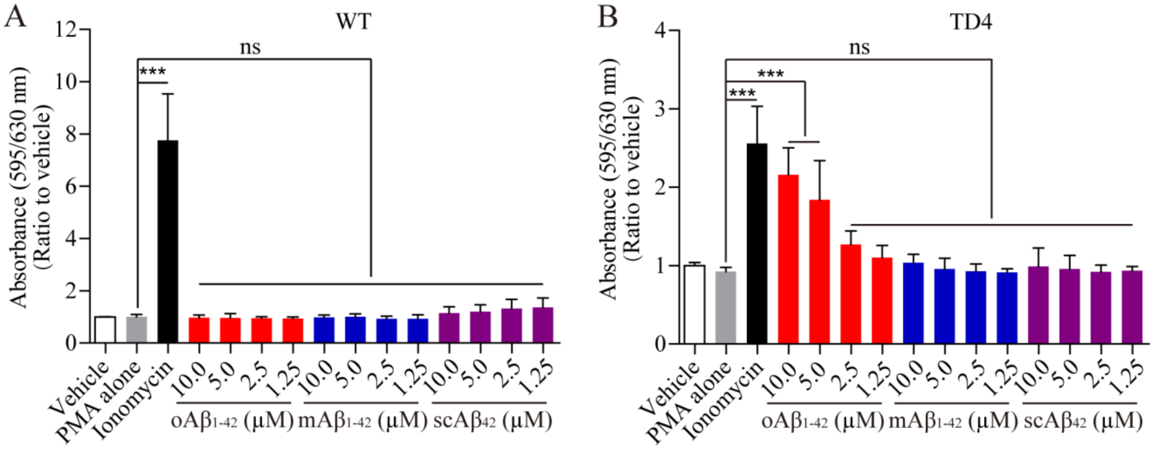

Fig. S2 Oligomeric A$\beta_{1\text{-}42}$ specifically activates TREM2 reporter cells. (A and B) WT (A) or TD4 BWZ reporter cells (B) were treated with oA$\beta_{1\text{-}42}$, mA$\beta_{1\text{-}42}$ or scAβ_{42} at the indicated concentrations with the presence of PMA. Ionomycin served as a positive control. After 16 hours, β-galactosidase activity was determined to assess the activity of TREM2-dependent signaling (at least 4 repeats per group, one-way ANOVA). Data information: Data represent mean ± SD. ***, $p < 0.001$; ns, not significant.

Figure S3

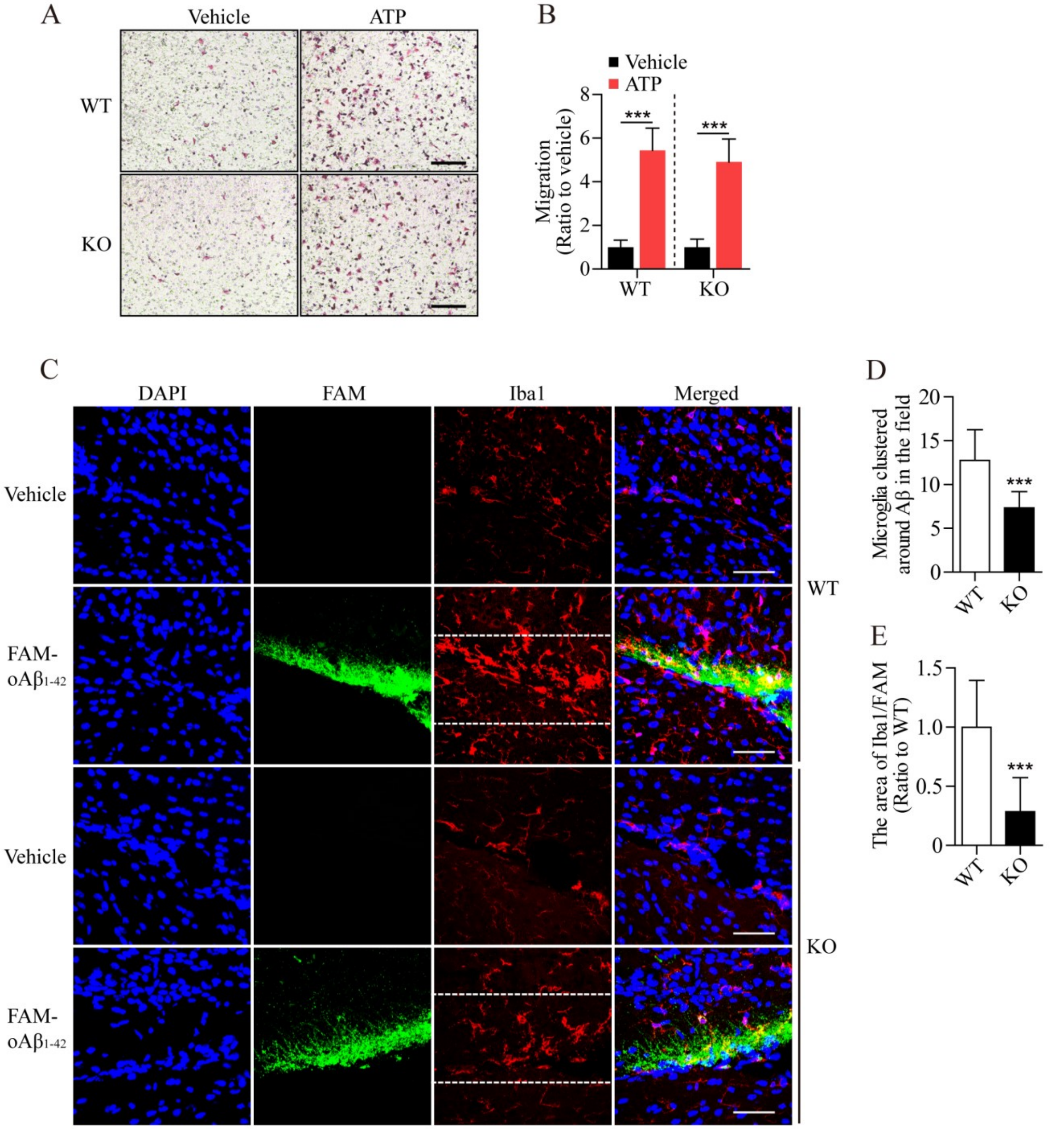

Fig. S3 The number of microglia clustered around Aβ is decreased in *Trem2*-KO mice. (A) Primary microglial cells (10^5) from WT or *Trem2*-knockout (KO) mice were plated onto transwell chamber inserts. Following 20 hours incubation with vehicle or ATP (60 µM), cells migrated through the membrane were stained with hematoxylin and eosin and imaged under Nikon inverted microscope. Scale bar, 100 µm. (B) At least 9 different fields from three independent

experiments of transwell tests were selected for quantifying the number of migrated cells (unpaired Student's *t*-test). (C) WT or *Trem2*-KO (KO) mice brain was harvested after FAM-oA$\beta_{1\text{-}42}$ injection for 16 hours. Coronal sections from these mice were stained with DAPI (blue) for nuclei, and Iba1 (red) for microglia. Representative z stack images of oA$\beta_{1\text{-}42}$-bearing regions are shown. Scale bar, 50 μm. (D) At least 12 fields (212 μm × 100 μm, marked by white dotted lines in the panel C from four mice were selected for quantifying the number of microglia (co-stained with DAPI and Iba1) clustered in the oA$\beta_{1\text{-}42}$-bearing regions (unpaired, Student's *t*-test). (E) The Iba1-positive area within the white dotted lines in the panel C was normalized to that of FAM-oA$\beta_{1\text{-}42}$-positive area (unpaired, Student's *t*-test). Data information: Data represent mean ± SD. ***, $p < 0.001$.
